# Association of serum 25-hydroxyvitamin D levels with aggressiveness of papillary thyroid cancer

**DOI:** 10.1530/EC-23-0373

**Published:** 2023-12-12

**Authors:** Yuting Shao, Xiaole Hu, Yuxi Wang, Yi Shao, Luchuan Li, Qingdong Zeng, Hong Lai, Lei Sheng

**Affiliations:** 1 Department of Endocrinology, Qilu Hospital of Shandong University, Jinan, Shandong, China; 2 Department of Operating Room, Qilu Hospital of Shandong University, Shandong, China; 3 Department of Breast and Thyroid Surgery, People’s Hospital of Mengyin County, Linyi, Shandong, China; 4 Department of Thyroid Surgery, General Surgery, Qilu Hospital of Shandong University, Jinan, Shandong, China

**Keywords:** 25-hydroxyvitamin D, papillary thyroid cancer, aggressiveness, lymph node metastasis, multifocality

## Abstract

**Objective:**

Serum 25-hydroxyvitamin D (25(OH)D) deficiency has been known to be associated with the risk and mortality of several cancers. However, the role of 25(OH)D in papillary thyroid cancer (PTC) remains controversial. This study aimed to investigate the association between 25(OH)D and clinicopathologic features of PTC.

**Methods:**

Patients who underwent thyroidectomy were retrospectively reviewed. Serum 25(OH)D levels were measured within a week prior to surgery. The patients were categorized into four quartiles according to season-specific 25(OH)D levels. The association between 25(OH)D levels and clinicopathologic features of PTC was analyzed.

**Results:**

A total of 2932 patients were enrolled in the study. The 25(OH)D levels were significantly higher in patients with lymph node metastasis (LNM; *P* < 0.001), lateral LNM (*P* < 0.001), and multifocal tumors (*P* < 0.001). Compared to the first quartile (Q1) of 25(OH)D level, the third quartile (Q3) and the fourth quartile (Q4) showed an unadjusted OR of 1.36 (95% CI: 1.09–1.69; *P* = 0.006) and 1.76 (95% CI: 1.42–2.19; *P* < 0.001) for LNM (*P* for trend < 0.001), respectively. An increased risk of multifocal tumors was strongly associated with high 25(OH)D concentration (*P* for trend <0.001). Similar results were obtained after adjusting for confounding factors.

**Conclusion:**

High 25(OH)D levels are associated with aggressive features of PTC, such as lymph node metastasis and multifocality.

## Introduction

The incidence of thyroid cancer (TC) has increased rapidly and has ranked the seventh in cancer incidence among female in the United States in 2023 (1). In China, thyroid cancer is the fourth most common malignancy in female, accounting for 37.36% of new cases of all types of cancer in 2020 (2). PTC accounts for 80% of the TC. This phenomenon can be partially attributed to the enhanced detection such as ultrasound and fine-needle aspiration biopsy, but it does exist because of the true increase of PTC incidence (3). Although the mortality rate of PTC is very low (4), its prognostic factors deserve attention because of the large population base of incidence.

25(OH)D, a kind of steroid hormone mainly produced in skin, regulates bone metabolism and calcium and phosphorus homeostasis. 25(OH)D deficiency is related to several diseases, including cardiovascular diseases such as hypertension and heart failure (5), insulin resistance (6), and autoimmune diseases such as rheumatoid arthritis (7). Recently, several studies have demonstrated that 25(OH)D deficiency is associated with the risk and mortality of cancer, including breast, prostate, and colorectal cancer (8, 9, 10, 11, 12, 13). In 2022, a historical overview showed a reverse correlation between serum 25(OH)D levels and the incidence of 12 types of cancer (14). However, the clinical evidence of an association between serum 25(OH)D levels and clinicopathological features of TC remains inconsistent. Kim *et al.* retrospectively investigated 548 female patients who underwent thyroidectomy for PTC and found that 25(OH)D levels were significantly lower in patients with a tumor size >1 cm or lymph node metastasis. Those with 25(OH)D values below the median had a significantly higher risk of T stage 3/4, LNM, lateral LNM, stage III/IV, and extrathyroidal extension (ETE) (15). Conversely, a study in 2022 revealed that low serum vitamin D levels were not associated with the aggressive pathological features such as multicentricity, lymphovascular invasion, and central or lateral LNM (16). These results were consistent with those of a study conducted in China (17). Furthermore, there were also meta-analyses supporting the hypothesis of an inverse correlation between 25(OH)D levels and prognosis of PTC (18, 19). A limitation of these studies is that most of them have small sample size. Hence, our study aimed to further evaluate the relationship between 25(OH)D levels and clinicopathologic features of PTC conducted in a relatively large population.

## Materials and methods

### Study population

We retrospectively reviewed the patients from December 2017 to December 2021 at Qilu hospital of Shandong University and the inclusion criteria were (i) patients who underwent thyroidectomy and (ii) patients diagnosed with thyroid cancer pathologically. The exclusion criteria were (i) TC patients with pathological types except PTC or with uncertain pathological types, (ii) patients under 18 years of age, (iii) patients with hyperparathyroidism, (iv) patients who had vitamin D supplementation within 3 months before surgery, and (v) patients with incomplete original data such as serum 25(OH)D or main clinicopathologic characters such as tumor size. Informed consent was obtained from all patients at the time of surgery. This study was approved by the ethics committees of Qilu Hospital of Shandong University (ethical code number: KYLL-2018 (KS)-055).

### Data acquisition

Since all patients were asked for the basic information and underwent blood tests within a week prior to surgery, the medical records were retrospectively reviewed from the electronic medical records system, including age, sex, height, weight, date of diagnosis, serum 25(OH)D, thyroid-stimulating hormone (TSH), phosphorus (P), calcium (Ca^2+^), parathyroid hormone (PTH), primary tumor size, multifocality, ETE, LNM, lateral lymph node metastasis, and Hashimoto’s thyroiditis.

### Statistical analysis

Statistical analyses were performed using IBM SPSS version 26.0 (IBM Corp.), GraphPad Prism 9 and R software package version 3.0. The NCCN guideline (version 3.2022) was referred to for the PTC stage (20). According to National Institutes of Health, vitamin D deficiency was defined by 25(OH)D levels ≤12 ng/mL and insufficiency by 25(OH)D levels from 13 to 20 ng/mL. Vitamin D sufficiency was defined by 25(OH)D levels >20 ng/mL (21). Because of the different distribution of 25(OH)D in different months, we analyzed our original data and found that 25(OH)D concentrations were notably higher in sunnier months (June–November) than in darker months (December–May) (Supplementary Fig. 1, see the section on supplementary materials given at the end of this article). Thus, 25(OH)D concentrations were categorized by quartile according to these specific seasons and then merged together. The continuous values in this study did not correspond to normal distribution according to the Kolmogorov–Smirnov test, so they were expressed as median value and interquartile ranges (IQR). Categorical values were reported as frequency and proportion (%). Wilcoxon rank-sum tests were performed for continuous variables and chi-square tests were conducted for categorical variables. Spearman’s correlation was performed to determine the relationship between 25(OH)D levels and other variables. We used univariate and multivariate logistic regression analyses to explore the role of 25(OH)D as a prognostic indicator of PTC. *P* value for trend was tested by assigning consecutive scores to the quartiles. Statistical significance was set at a two-sided *P* < 0.05.

## Results

**Figure 1:**
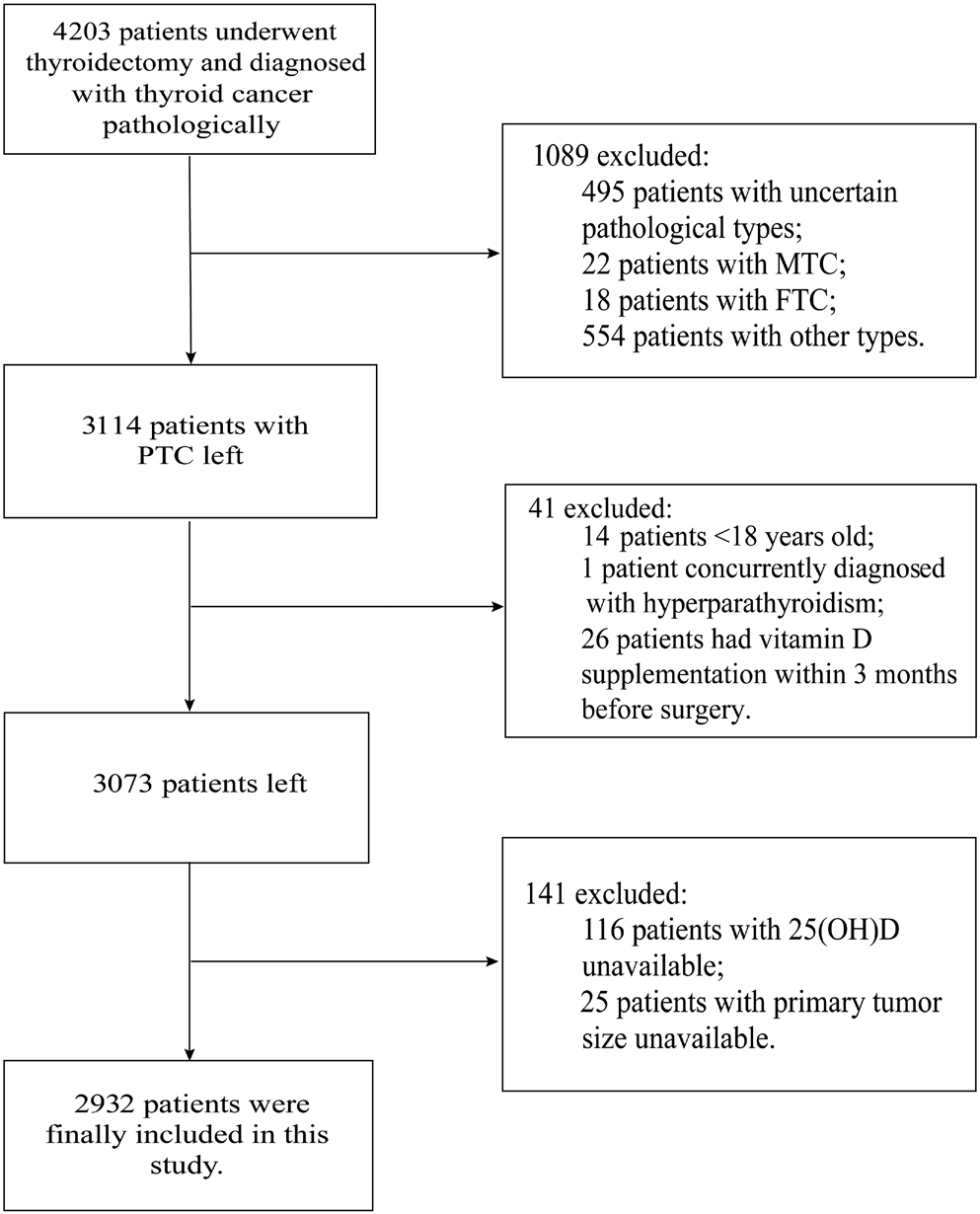
Flowchart of patient selection.

In total, 4177 individuals diagnosed with thyroid cancer were retrospectively reviewed. After strict selection based on inclusion and exclusion criteria, a total of 2932 patients (2235 females and 637 males) were eligible for this study. A flowchart of patient selection was shown in Fig. 1. The clinical characteristics of the included patients were shown in Table 1. The age ranged from 18 to 80 years (median (IQR): 45(18)). Most patients with PTC had either vitamin D insufficiency (46.0%) or deficiency (19.6%), while only 34.4% of patients had vitamin D sufficiency. The 25(OH)D level was significantly higher in patients aged ≥55 years than in those aged <55 years (18.91 vs 16.40 ng/mL, *P* < 0.001) and in males than in females (22.43 vs 17.22 ng/mL, *P* < 0.001). Unexpectedly, as the LNM stage increased, the serum 25(OH)D levels also increased (*P* < 0.001). Patients with lateral LNM displayed higher 25(OH)D levels than those without (18.52 vs 16.80 ng/mL, *P* < 0.001). Moreover, 25(OH)D levels were higher in patents with stage II/III tumors than in those with stage I tumors (*P* < 0.001), and were higher in patients with multifocal tumors than in those with unifocal tumor (*P* < 0.001). Moreover, patients with concurrent Hashimoto’s thyroiditis had lower 25(OH)D levels than those without concurrent thyroiditis. There was no statistical difference in the 25(OH)D levels in terms of tumor size and T stage. Although 25(OH)D levels showed a decreased trend in patients with ETE, this difference was not statistically significant.

**Table 1:** Relationship between 25(OH)D levels and the clinicopathologic characteristics of PTC.

Variable	Number of patients (%)	25(OH)D (ng/mL, IQR)	*P*
Age			<0.001
<55 years	2272 (77.5)	16.40 (8.90)	
≥55 years	660 (22.5)	18.91 (10.82)	
Sex			<0.001
Female	2235 (76.2)	16.00 (8.54)	
Male	697 (23.8)	20.79 (11.34)	
Month			<0.001
Darker month (December–May)	1580 (53.9)	14.88 (7.57)	
Sunnier month (June–November)	1352 (46.1)	19.13 (10.24)	
25(OH)D status			<0.001
Deficiency	574 (19.6)	10.06 (2.44)	
Insufficiency	1348 (46.0)	15.78 (3.88)	
Sufficiency	1010 (34.4)	24.96 (7.80)	
Tumor size			0.057
≤1 cm	2072 (70.7)	16.74 (9.23)	
>1 cm	860 (29.3)	17.59 (10.26)	
T stage			0.874
1/2	1189 (40.6)	16.90 (9.63)	
3/4	1743 (59.4)	17.12 (9.27)	
N stage			<0.001
N0	1936 (66.1)	16.39 (8.65)	
N1a	643 (21.9)	18.02 (10.81)	
N1b	352 (12.0)	18.52 (10.40)	
Lateral LNM			<0.001
Negative	2580 (88.0)	16.80 (9.30)	
Positive	352 (12.0)	18.52 (10.40)	
Stage			<0.001
I	2486 (84.8)	16.54 (8.95)	
II	409 (13.9)	19.04 (10.86)	
III	37 (1.3)	19.19 (15.30)	
Multifocality			<0.001
Negative	1878 (64.1)	16.61 (9.19)	
Positive	1054 (35.9)	17.53 (10.19)	
Bilateral			<0.001
Negative	2263 (77.2)	16.70 (9.30)	
Positive	669 (22.8)	17.91 (10.34)	
Hashimoto’s thyroiditis			<0.001
Negative	2279 (77.7)	17.28 (9.51)	
Positive	646 (22.3)	16.13 (9.28)	
ETE			0.951
Negative	1194 (40.7)	16.90 (9.67)	
Positive	1738 (59.3)	17.12 (9.23)	

25(OH)D, 25-hydroxyvitamin D; ETE, extrathyroidal extension; IQR, interquartile range; LNM, lymph node metastasis.

Table 2 shows the clinicopathologic characteristics according to the 25(OH)D quartile. The percentage of males was higher in quartile 4 than in the other quartiles (*P* < 0.001). Proportion of patients who were with lymph node metastasis increased from the first quartile to the fourth quartile (29.3%, 28.2%, 36.0%, 42.2%; *P* < 0.001). Similar trend was also observed in proportion of patients who had lateral LNM (9.4%, 10.9%, 12.8%, 14.9%; *P* = 0.009) and patients who had multifocal tumors (30.7%, 34.0%, 39.0%, 40.1%; *P* < 0.001).

**Table 2:** Clinicopathologic characteristics of PTC according to quartiles of serum 25(OH)D.

Variables	Total (*N* = 2932)	Quartile 1 (*N* = 733)	Quartile 2 (*N* = 733)	Quartile 3 (*N* = 733)	Quartile 4 (*N* = 733)	*P*
25(OH)D, ng/mL, median (IQR)	17.01 (9.44)	10.67 (3.17)	15.12 (3.93)	19.43 (5.29)	27.43 (8.81)	<0.001
Age, years, median (IQR)	45 (18.00)	42 (17.00)	43 (17.00)	45 (18.00)	49 (16.00)	<0.001
Sex, male, *n* (%)	697 (23.8)	48 (8.9)	96 (15.5)	152 (22.1)	401 (36.9)	<0.001
BMI, kg/m^2^, median (IQR)	24.77 (4.86)	24.1 (5.27)	24.63 (5.09)	24.81 (4.69)	25.39 (4.53)	<0.001
Tumor size, cm, median (IQR)	0.8 (0.70)	0.7 (0.60)	0.8 (0.60)	0.8 (0.70)	0.8 (0.70)	0.247
Stage III/IV, *n* (%)	37 (1.3)	11 (1.5)	3 (0.4)	9 (1.2)	14 (1.9)	0.680
LNM, *n* (%)	995 (33.9)	215 (29.3)	207 (28.2)	264 (36.0)	409 (42.2)	<0.001
Lateral LNM, *n* (%)	352 (12.0)	69 (9.4)	80 (10.9)	94 (12.8)	109 (14.9)	0.009
Multifocality, *n* (%)	1054 (35.9)	225 (30.7)	249 (34.0)	286 (39.0)	294 (40.1)	<0.001
Bilateral, *n* (%)	669 (22.8)	146 (19.9)	146 (19.9)	180 (24.6)	197 (26.9)	0.002
Hashimoto’s thyroiditis, *n* (%)	646 (22.1)	174 (23.9)	179 (24.5)	146 (19.9)	247(20.1)	0.205
PTH, pg/mL, median (IQR)	38.77 (18.98)	42.23 (19.69)	39.35 (19.45)	37.50 (17.68)	33.88 (17.22)	<0.001
Ca^2+^, mmol/L, median (IQR)	2.33 (0.13)	2.31 (0.13)	2.33 (0.12)	2.33 (0.12)	2.36 (0.13)	<0.001
P, mmol/L, median (IQR)	1.18 (0.22)	1.19 (0.22)	1.18 (0.20)	1.16 (0.23)	1.15 (0.22)	0.011
TSH, mIU/L, median (IQR)	1.75 (1.39)	1.76 (1.37)	1.78 (1.33)	1.71 (1.22)	1.69 (1.46)	0.828
ETE, n (%)	1738 (59.3)	432 (58.9)	434 (59.2)	430 (58.7)	442 (60.3)	0.926

25(OH)D, 25 hydroxyvitamin D; BMI, body mass index; Ca^2+^, calcium; IQR, interquartile range; ETE, extrathyroidal extension; LNM, lymph node metastasis; P, phosphorus; PTH, parathyroid hormone; TSH, thyroid-stimulating hormone.

We performed Spearman’s correlation to analysis the relationship between prognostic factors and 25(OH)D levels (Supplementary Table 1). Positive correlations weakly exist between age, BMI, calcium, LNM, stage and serum 25(OH)D levels. In contrast, PTH and phosphorus levels were inversely correlated with serum 25(OH)D levels. All the differences were statistically significant (*P* < 0.001).

To evaluate the effect of 25(OH)D on the aggressive features of PTC, logistic regression analyses were performed for each quartile of serum 25(OH)D levels (Table 3). The first quartile of 25(OH)D level was established as a reference. Compared to the first quartile, the third quartile and the fourth quartile showed an unadjusted OR of 1.36 (95% Cl: 1.09–1.69; *P* = 0.006) and 1.76 (95% Cl: 1.42–2.19; *P* < 0.001) for LNM (*P* for trend < 0.001), respectively. Similarly, Q3 and Q4 showed an unadjusted OR of 1.42 (95% Cl 1.02–1.97; *P* = 0.038) and 1.68 (95% Cl 1.22–2.32; *P* = 0.002) for lateral LNM (*P* for trend <0.001), respectively. An increased risk of multifocal tumors was strongly associated with high serum 25(OH)D concentration (Q4 vs Q1, OR 1.51, 95% CI 1.22–1.88, *P* < 0.001; Q3 vs Q1, OR 1.45, 95% CI 1.16–1.79, *P* = 0.001; *P* for trend <0.001). In addition, the risk of tumor size >1 cm also increased when 25(OH)D level was in quartile 4.

**Table 3:** Logistic regression analysis of the effect of 25(OH)D on the aggressiveness of PTC.

Variables	Quartile 1	Quartile 2		Quartile 3		Quartile 4		*P*for trend
	OR	OR	*P*	OR	*P*	OR	*P*	
Multifocal	1 (Ref)	1.16 (0.93, 1.45)	0.180	1.45 (1.16, 1.79)	0.001	1.51 (1.22, 1.88)	<0.001	<0.001
Bilateral	1 (Ref)	1.00 (0.77, 1.29)	1.000	1.31 (1.02, 1.68)	0.033	1.48 (1.16, 1.89)	0.002	<0.001
Tumor size >1 cm	1 (Ref)	0.95 (0.75, 1.19)	0.640	1.07 (0.85, 1.34)	0.564	1.28 (1.02, 1.60)	0.031	0.011
T stage III/IV	1 (Ref)	1.01 (0.82, 1.25)	0.915	0.98 (0.80, 1.21)	0.873	1.07 (0.87, 1.32)	0.523	0.538
LNM	1 (Ref)	0.95 (0.76, 1.19)	0.644	1.36 (1.09, 1.69)	0.006	1.76 (1.42, 2.19)	<0.001	<0.001
Lateral LNM	1 (Ref)	1.18 (0.84, 1.66)	0.342	1.42 (1.02, 1.97)	0.038	1.68 (1.22, 2.32)	0.020	0.001
Stage III/IV	1 (Ref)	0.27 (0.08, 0.97)	0.045	0.82 (0.34, 1.98)	0.653	1.28 (0.58, 2.83)	0.546	0.187
ETE	1 (Ref)	1.01 (0.82, 1.25)	0.915	0.99 (0.80, 1.22)	0.915	1.06 (0.86, 1.30)	0.595	0.633

Cut points for season-specific quartile (ng/mL): darker months (December–May) = quartile 1: ≤9.56, quartile 2: >9.56 and ≤12.2, quartile 3: >12.2 and ≤15.6, quartile 4: >15.6; sunnier months (June–November) = quartile 1: ≤14.4, quartile 2: >14.4 and ≤18.76, quartile 3: >18.76 and ≤23.44, quartile 4: >23.44.The first quartile of 25(OH)D was established as reference.

ETE, extrathyroidal extension; LNM, lymph node metastasis; OR, odds ratio.

After adjusting for age, sex, and BMI (Table 4), the risk of lymph node metastasis and lateral LNM increased by 75% (OR 1.75, 95% CI 1.39–2.21, *P* < 0.001) and 56% (OR 1.56, 95% CI 1.11–2.20, *P* = 0.01) with Q4 of 25(OH)D levels, respectively. Likewise, patients with Q4 of 25(OH)D levels were more likely to have multifocal tumors compared with Q1 (OR 1.43, 95% CI 1.17–1.80, *P* = 0.002). A similar trend was also observed after further adjustment for calcium, phosphorus, TSH, and Hashimoto’s thyroiditis. Previous studies have found that tumor multifocality is an independent risk factor for both central and lateral lymph node metastasis of PTC (22, 23, 24, 25). Based on this, we further adjusted for multifocality, together with other variables in model 3. To our surprise, higher 25(OH)D levels (Q4) remained an independent risk factor for LNM (OR 1.90, 95% CI 1.36–2.67, *P* < 0.001; *P* for trend <0.001), indicating the robustness and reliability of our results.

**Table 4:** Logistic regression analysis of the effect of 25(OH)D on the aggressiveness of PTC after adjusting factors.

Variables	Model 1		Model 2		Model 3	
	OR (95% CI)	*P*	OR (95% CI)	*P*	OR (95% CI)	*P*
Multifocal						
Quartile 1	1 (Ref)		1 (Ref)		N/A	N/A
Quartile 2	1.15 (0.92–1.44)	0.206	1.19 (0.90–1.58)	0.226	N/A	N/A
Quartile 3	1.43 (1.14–1.78)	0.002	1.32 (0.99–1.75)	0.063	N/A	N/A
Quartile 4	1.43 (1.14–1.80)	0.002	1.53 (1.13–2.09)	0.007	N/A	N/A
* P* for trend		<0.001		0.005		N/A
Bilateral						
Quartile 1	1 (Ref)		1 (Ref)		1 (Ref)	
Quartile 2	0.99 (0.77–1.29)	0.964	1.08 (0.78–1.49)	0.652	0.88 (0.56–1.40)	0.594
Quartile 3	1.28 (1.00–1.65)	0.053	1.36 (0.98–1.89)	0.065	1.24 (0.77–1.99)	0.375
Quartile 4	1.39 (1.07–1.80)	0.014	1.71 (1.21–2.40)	0.002	1.53 (0.93–1.02)	0.097
* P* for trend		0.004		<0.001		0.033
Tumor size > 1cm						
Quartile 1	1 (Ref)		1 (Ref)		1 (Ref)	
Quartile 2	0.95 (0.75–1.19)	0.645	0.93 (0.68–1.26)	0.623	0.90 (0.66–1.23)	0.499
Quartile 3	1.06 (0.84–1.33)	0.638	1.05 (0.77–1.43)	0.772	1.00 (0.73–1.37)	0.993
Quartile 4	1.26 (1.00–1.60)	0.053	1.27 (0.92–1.77)	0.152	1.20 (0.86–1.67)	0.292
* P* for trend		0.023		0.075		0.223
LNM						
Quartile 1	1 (Ref)		1 (Ref)		1 (Ref)	
Quartile 2	0.95 (0.75–1.19)	0.627	0.99 (0.72–1.37)	0.972	0.96 (0.69–1.32)	0.779
Quartile 3	1.35 (1.08–1.69)	0.009	1.38 (1.00–1.89)	0.048	1.31 (0.95–1.80)	0.099
Quartile 4	1.75 (1.39–2.21)	<0.001	2.03 (1.45–2.83)	<0.001	1.90 (1.36–2.67)	<0.001
* P* for trend		<0.001		<0.001		<0.001
Lateral LNM						
Quartile 1	1 (Ref)		1 (Ref)		1 (Ref)	
Quartile 2	1.15 (0.82–1.62)	0.426	1.04 (0.68–1.58)	0.865	0.97 (0.63–1.48)	0.869
Quartile 3	1.36 (0.97–1.90)	0.074	1.11 (0.73–1.70)	0.631	0.99 (0.64–1.53)	0.964
Quartile 4	1.56 (1.11–2.20)	0.010	1.53 (0.99–2.38)	0.058	1.35 (0.86–2.12)	0.189
* P* for trend		<0.001		0.037		0.184
Stage III/IV						
Quartile 1	1 (Ref)		1 (Ref)		1 (Ref)	
Quartile 2	0.35 (0.10–1.26)	0.083	0.18 (0.02–1.66)	0.130	1.17 (0.89–1.56)	0.263
Quartile 3	0.95 (0.39–2.34)	0.740	1.06 (0.29–3.92)	0.928	0.88 (0.66–1.18)	0.394
Quartile 4	0.89 (0.37–2.16)	0.800	0.62 (0.16–2.39)	0.487	1.10 (0.80–1.51)	0.548
* P* for trend		0.744		0.789		0.924
ETE						
Quartile 1	1 (Ref)		1 (Ref)		1 (Ref)	
Quartile 2	1.00 (0.81–1.23)	0.996	1.16 (0.88–1.53)	0.281	1.17 (0.89–1.55)	0.268
Quartile 3	0.97 (0.78–1.20)	0.763	0.92 (0.69–1.21)	0.535	0.89 (0.67–1.89)	0.433
Quartile 4	1.00 (0.80–1.24)	0.983	1.10 (0.81–1.49)	0.545	1.08 (0.79–1.47)	0.653
*P* for trend		0.904		0.993		0.838

Cut points for season-specific quartile (ng/mL): darker months (December–May) = quartile 1: ≤9.56, quartile 2: >9.56 and ≤12.2, quartile 3: >12.2 and ≤15.6, quartile 4: >15.6; sunnier months (June–November) = quartile 1: ≤14.4, quartile 2: >14.4 and ≤18.76, quartile 3: >18.76 and ≤23.44, quartile 4: >23.44.The first quartile of 25(OH)D was established as reference. Model 1 was adjusted for age, sex, and BMI. Model 2 was adjusted for age, sex, BMI, PTH, Ca, P, and Hashimoto’s thyroiditis. Model 3 was adjusted for age, sex, BMI, PTH, Ca, P, Hashimoto’s thyroiditis, and multifocality.

ETE, extrathyroidal extension; LNM, lymph node metastasis; OR, odds ratio.

## Discussion

In this study, we performed several statistical analyses to explore the relationship between 25(OH)D levels and clinicopathological features of PTC. We divided the patients into four quartiles according to season-adjusted 25(OH)D levels. Surprisingly, the 25(OH)D levels were significantly higher in patients with lymph node metastasis, lateral LNM, and multifocal tumors. A greater proportion of multifocality and higher risk of LNM were found in the fourth quartile of 25(OH)D levels. This finding was inconsistent with the widely held assumption that 25(OH)D deficiency was related to advanced cancer stage and increased incidence of metastasis and recurrence (26, 27, 28).

Kim *et al.* found that patients in the second lowest quartile of 25(OH)D had a greater occurrence of LNM (OR 2.03, 95% CI 1.19–3.44, *P* = 0.009) and lateral LNM (OR 5.12, 95% CI 1.68–15.59, *P* = 0.009) than those in the highest quartile (15). Cocolos *et al.* described histopathologic features of 170 patients with differentiated thyroid cancer and found patients in T stage 4 had significantly lower 25(OH)D of 10.96 ng/mL than in T stage 1 of 18.24 ng/mL, suggesting that patients with aggressive tumors had lower circulating levels of 25(OH)D (29). On the contrary, Demircioglu *et al*., Kuang *et al.* and Ahn *et al.* showed serum 25(OH)D levels were not associated with disease aggressiveness such as LNM, tumor size, lateral LNM, or multifocality (16, 17, 30).

Given that numerous epidemiological and experimental data have demonstrated that vitamin D can inhibit tumor growth and metastasis, we can only speculate on the possible causes of higher 25(OH)D levels associated with lymph node metastasis and multifocality of tumors without further basic research. 1,25-dihydroxyvitamin D (1,25(OH)_2_D), transformed by 25(OH)D, has been proven to have anti-tumor effect by its antiproliferative and redifferentiation capacity (31, 32). 1,25(OH)_2_D plays its biological role through binding to the vitamin D receptor (VDR), which belongs to the nuclear receptor family. 1,25(OH)_2_D is inactivated by 24-hydroxylase (CYP24A1) (33). Clinckspoor *et al.* found that in PTC with lymph node metastasis, VDR was decreased than that in nonmetastasized PTC (34). Furthermore, VDR expression was often lost in anaplastic thyroid cancer (ATC). Besides, ATC with high ki67 expression (>30%) or distant metastases was characterized by more negative VDR staining. Therefore, it is reasonable to infer that in tumors with lymph node metastasis, owing to the reduced or absent VDR expression, although with a high level of 1,25(OH)_2_D, they cannot sufficiently bind to VDR and exert its anti-tumor effects. In our previous study, we found that in malignant tissue, there was significant induction of the expression of the CYP24A1 gene following treatment with 1,25(OH)_2_D, suggesting that *CYP24A1* was target gene of 1,25(OH)_2_D (35). Clinckspoor *et al.* also found CYP24A1 expression was decreased in PTC with LNM. This may be due to either reduced expression of VDR or decreased local availability of 1,25(OH)_2_D within the tumor microenvironment. In the context of our study, impaired VDR signaling pathway and decreased 1,25(OH)_2_D levels within tumor microenvironment may coexist to limit its antitumor effect in patients with LNM, even if the serum 25(OH)D level was high. Unfortunately, local 1,25(OH)_2_D levels were not measured in our study.

In the liver, vitamin D is metabolized by vitamin D 25-hydroxylase (CYP2R1 and CYP27A1) to 25(OH)D. 25(OH)D is further metabolized by 25(OH)D-1alpha-hydroxylase (CYP27B1) mainly in the proximal tubule of the kidney to 1,25(OH)2D, which is the most biologically active form of vitamin D (36, 37, 38). Several studies have demonstrated that CYP27B1 gene is underexpressed in tumor tissues including PTC, especially in metastases (34, 39, 40, 41). The reduction of CYP27B1 leads to reduced local transformation of 25(OH)D to 1,25(OH)2D in PTC patients with LNM, which may partially contribute to the elevation of serum 25(OH)D levels. Another possible explanation for the high serum 25(OH)D levels in patients with LNM is that PTC with aggressive features may secrete some circulating factors to cause impaired metabolic activity of vitamin D enzymes in kidney or liver contributing to elevated serum 25(OH)D levels, which warrants further investigation.

The strength of our study is that a large number of patients were included, which can provide more reliable evidence for the effect of 25(OH)D levels on the aggressiveness of PTC. Furthermore, because the months of 25(OH)D measured varied and the distribution of 25(OH)D differed by months, we categorized 25(OH)D levels by the sunnier and darker months and grouped the population according to the season-adjusted 25(OH)D levels to avoid the seasonal difference.

Our study still has several limitations. First, we used a cross-sectional design and enrolled patients only from a single center. Second, PTC is a kind of relatively indolent cancer with a favorable long-term survival rate, thus this study was short of a longer follow-up duration to observe the long-term prognosis of PTC. Third, we just have a single evaluation of 25(OH)D level before surgery, which does not fully represent one’s dynamic vitamin D status. Finally, although we attempted to speculate on the reason why the 25(OH)D levels have positive relations with aggressiveness factors of PTC, the specific molecular mechanism behind this phenomenon could not be determined due to the lack of further basic experimental exploration.

## Conclusion

High 25(OH)D levels are positively correlated with aggressive features of PTC, such as lymph node metastasis and multifocality. Randomized clinical trials with a long follow-up duration are required to establish the role of 25(OH)D in the long-term prognosis of PTC.

## Supplementary Materials

Supplementary Material

## Declaration of interest

The authors declare that the study was conducted in the absence of any commercial or financial relationships that could be construed as a potential conflict of interest.

## Funding

This study was supported by the National Natural Science Foundation of Chinahttp://dx.doi.org/10.13039/501100001809 (grant no. 81802642; grant recipient: Lei Sheng), Key Research and Developmenthttp://dx.doi.org/10.13039/100006190 Plan of Shandong Province (grant no. 2019GSF108099; grant recipient: Hong Lai), and Beijing Health Promotion Associationhttp://dx.doi.org/10.13039/100020440 (grant no. 6010122203; grant recipient: Lei Sheng).

## Ethical approval

This study was approved by the ethics committees of Qilu Hospital of Shandong University.

## Author contribution statement

L.S. conceived the study and its design. Y.T.S. and X.L.H. conducted data collection, statistical analysis, and manuscript drafting. Y.X.W., Y.S., L.C.L., Q.D.Z., and H.L. helped with the study design. All authors approved the final version of the manuscript.
