# Supplementary material for: Association of serum 25-hydroxyvitamin D levels with aggressiveness of papillary thyroid cancer
Source: Endocr Connect. 2023 Dec 12;13(1):e230373. doi: 10.1530/EC-23-0373 (PMC10762552; doi:10.1530/EC-23-0373)
Supplement: Supplementary Material [file supplementary_material.pdf]

Association of serum 25-hydroxyvitamin D levels with aggressiveness of papillary thyroid cancer

Yuting Shao, Xiaole Hu, Yi Shao, Luchuan Li, Qingdong Zeng, Hong Lai, Lei Sheng*

*** Correspondence:**

Corresponding Author: Dr. Lei Sheng

mail: lei.sheng@sdu.edu.cn

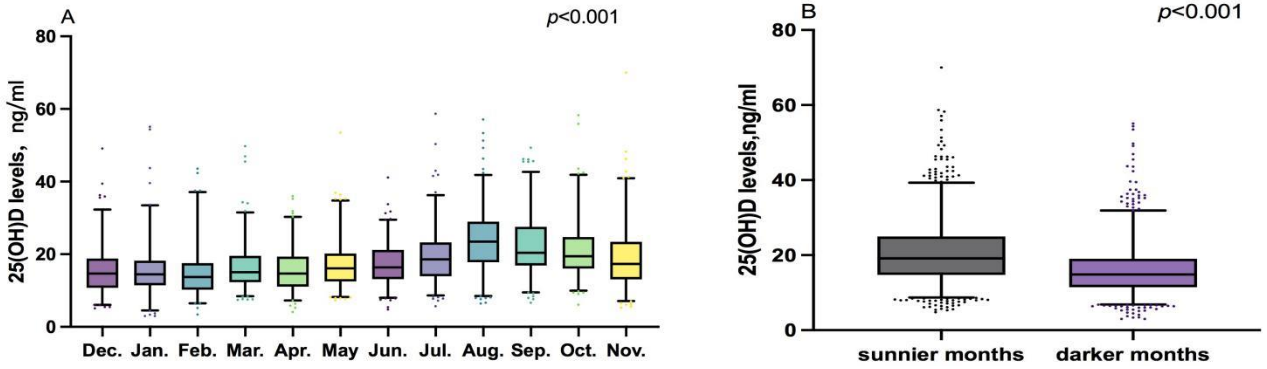

Supplementary Figure 1. 25(OH)D levels in 12 months (A) or in sunnier months (June to November) and darker months (December to May) (B). Statistic was performed by Wilcoxon rank sum tests. 25(OH)D concentrations were higher in sunnier months than in darker months (P<0.001).

Supplementary Table 1. Regression analysis between 25(OH)D and clinical features of PTC

Variables	N	r	*p* value
Age	2932	0.185	<0.001
BMI	2925	0.112	<0.001
PTH	2804	-0.237	<0.001
Ca^{2+}	2909	0.148	<0.001
P	1832	-0.090	<0.001
TSH	2852	-0.026	0.160
Tumor size	2392	0.031	0.098
N stage	2391	0.099	<0.001
Stage	2392	0.131	<0.001

BMI: Body Mass Index; PTH: parathyroid hormone; Ca2+, calcium; P, phosphorus; TSH: thyroid stimulating hormone
